# Concomitant Meckel’s Diverticulum With Focal Paneth Cell Metaplasia and Acute Appendicitis Presenting as Chronic Periumbilical Pain in a Child

**DOI:** 10.1155/cris/8396874

**Published:** 2026-10-08

**Authors:** Aditya Pachwa, Chaitanya Laasya Reddy Arimanda, Agasthya Daggu, Lakshya Nehal Samineni

**Affiliations:** ^1^ Mamata Academy of Medical Sciences Hyderabad 500118 India; ^2^ Malla Reddy Institute of Medical Sciences Hyderabad 500118 India; ^3^ Mamata Academy of Medical Sciences Hyderabad 500055 India; ^4^ College of Medical Sciences Teaching Hospital Chitwan Bharatpur 44200 Nepal

**Keywords:** abdominal pain, appendicitis, laparoscopy, Meckel diverticulum, Paneth cells

## Abstract

Meckel’s diverticulum is the most common congenital anomaly of the gastrointestinal tract but remains diagnostically challenging in children because of its varied and nonspecific clinical presentation. Concomitant Meckel’s diverticulum and acute appendicitis are uncommon and may further obscure diagnosis. We report a 9‐year‐old boy who presented with chronic periumbilical abdominal pain of 3 months’ duration, with acute worsening over 3 days accompanied by vomiting. Physical examination revealed localized right periumbilical tenderness without signs of peritonitis. Laboratory investigations demonstrated mild anemia and eosinophilia, while ultrasonography identified an appendicolith with mesenteric lymphadenitis, raising suspicion for appendiceal pathology. Owing to persistent symptoms and concern for evolving subacute intestinal obstruction, diagnostic laparoscopy was performed. Intraoperatively, acute appendicitis was identified along with a dilated Meckel’s diverticulum ~100 cm proximal to the ileocecal junction and associated proximal small‐bowel dilatation. Laparoscopic‐assisted Meckel’s diverticulectomy with appendectomy was successfully performed. Histopathologic examination confirmed acute appendicitis with a fecolith and transmural inflammatory infiltrates. The Meckel’s diverticulum demonstrated focal Paneth cell metaplasia and lymphoid aggregates consistent with Peyer patches without ectopic gastric or pancreatic tissue or atypia. The mesenteric lymph node showed reactive lymphoid hyperplasia. The postoperative course was uneventful, and the child remained symptom‐free at 2‐month follow‐up. This case highlights the importance of considering dual intraabdominal pathology in children presenting with prolonged or atypical abdominal pain. Diagnostic laparoscopy plays a pivotal role by enabling comprehensive abdominal evaluation and definitive treatment in a single setting. Furthermore, symptomatic Meckel’s diverticulum may occur in the absence of heterotopic mucosa, and uncommon histopathologic findings such as focal Paneth cell metaplasia underscore the value of meticulous examination of resected specimens.

## 1. Introduction

Meckel’s diverticulum is the most common congenital anomaly of the gastrointestinal tract but is often asymptomatic and difficult to diagnose preoperatively in children. Although many patients remain asymptomatic, symptomatic Meckel’s diverticulum in children may present with bleeding, diverticulitis, perforation, or intestinal obstruction, and its manifestations often overlap with other causes of acute abdomen [1, 2]. In pediatric practice, chronic or recurrent periumbilical and right‐sided abdominal pain may reflect appendicitis, Meckel’s diverticulum, mesenteric adenitis, intussusception, or other mimics, making accurate preoperative diagnosis difficult [2, 3]. Recent pediatric data further suggest that complicated Meckel’s diverticulum may closely simulate acute appendicitis, particularly when the presentation is dominated by abdominal pain and vomiting rather than gastrointestinal bleeding [3]. Diagnostic laparoscopy is therefore especially valuable when symptoms are prolonged, atypical, or disproportionate to preoperative findings because it permits complete abdominal inspection and definitive treatment during the same procedure [4, 5]. Rare reports of concurrent appendicitis and Meckel’s diverticular pathology further emphasize the importance of systematic inspection of the distal ileum even when appendiceal disease has already been identified [6]. We report a 9‐year‐old boy with chronic periumbilical pain and acute worsening, initially evaluated as appendiceal pathology with mesenteric lymphadenitis and possible evolving subacute intestinal obstruction, in whom diagnostic laparoscopy revealed a dilated Meckel’s diverticulum with proximal small bowel dilatation in addition to acute appendicitis; histopathologic examination further demonstrated focal Paneth cell metaplasia within the diverticulum.

This case is unique due to the combination of chronic abdominal pain, simultaneous appendicitis and Meckel’s diverticulum, and the histologic finding of focal Paneth cell metaplasia, which is rarely reported.

## 2. Case Presentation

A 9‐year‐old boy presented with abdominal pain of 3 months’ duration, insidious in onset and gradually progressive, with marked worsening over the preceding 3 days. The pain was centered in the umbilical and right periumbilical region. There was no vomiting during the initial 3 months; however, after the recent aggravation of pain, he developed 2 episodes of vomiting. There was no history of fever, gastrointestinal bleeding, constipation, diarrhea, or other altered bowel habits. His past medical history was unremarkable.

On examination, he was afebrile (97.6°F), with a pulse rate of 123/min, blood pressure of 112/80 mmHg, respiratory rate of 20/min, and oxygen saturation of 98% on room air. He weighed 20 kg and measured 124 cm in height. The abdomen was soft, with localized tenderness in the right periumbilical/umbilical region, without guarding, rigidity, palpable mass, organomegaly, or clinically evident distension.

Laboratory investigations showed hemoglobin 10 g/dL, RBC count 5.7 million/µL, white blood cell count 7700/mm^3^, eosinophils 8%, lymphocytes 46%, neutrophils 39%, serum creatinine 0.3 mg/dL, and adequate platelet count. Ultrasonography demonstrated a 5.5‐mm appendicolith with mesenteric lymphadenitis. In view of persistent abdominal pain, recent vomiting, and concern for appendiceal pathology with evolving subacute intestinal obstruction, diagnostic laparoscopy was undertaken.

Intraoperatively, the appendix was dilated at the tip with surrounding fat stranding. On further exploration, a dilated Meckel’s diverticulum was identified ~100 cm proximal to the ileocecal junction, with associated dilatation of proximal small bowel loops. Multiple mesenteric lymph nodes measuring less than 1 cm were also noted. No free intraperitoneal fluid, perforation, bowel ischemia, or gross contamination was present. Laparoscopic Meckel’s diverticulectomy with appendectomy was performed (Figures 1 and 2).

**Figure 1:**
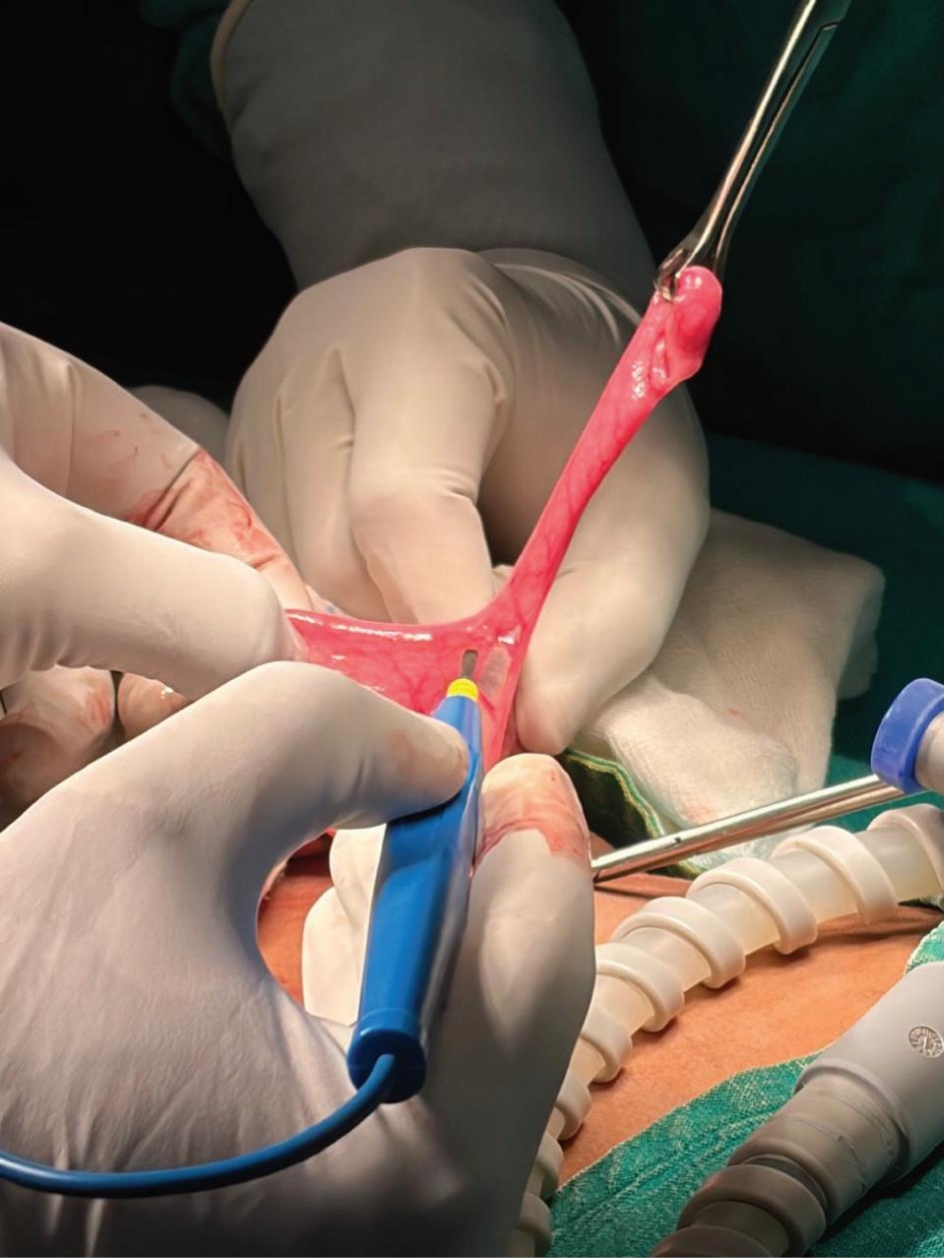
Intraoperative photograph showing exteriorized Meckel’s diverticulum during laparoscopic‐assisted resection.

**Figure 2:**
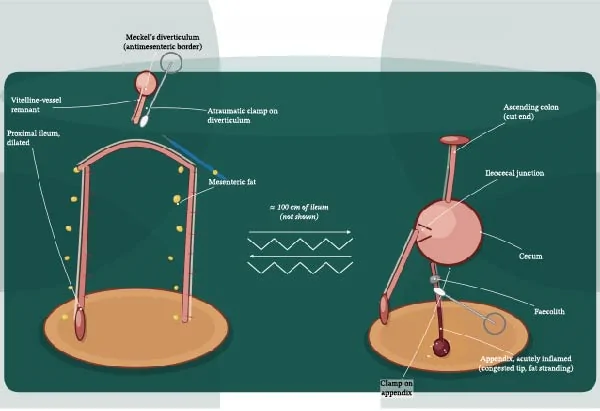
Intraoperative schematic: Meckel’s diverticulum and acute appendicitis. Surgery‐oriented illustrative reconstruction of the operative findings (not to scale). Left: the dilated Meckel’s diverticulum, arising from the antimesenteric border of the ileum and grasped with an atraumatic clamp, as exteriorized at operation. Right: the acutely inflamed, faecolith‐containing appendix at the cecum. The two findings, located ~100 cm apart along the same limb of bowel, are shown together to illustrate their coexistence and spatial relationship; the intervening ileum is omitted for clarity.

Histopathologic examination of the Meckel’s diverticulum specimen showed small‐intestinal mucosa lined by tall columnar epithelium with focal Paneth cell metaplasia and lymphoid aggregates consistent with Peyer patches. The muscularis propria and serosa were unremarkable, and no ectopic gastric or pancreatic tissue or atypia was identified. Histologic examination of the mesenteric lymph node showed preserved architecture with prominent germinal centers, consistent with reactive lymphoid hyperplasia (Figure 3). The appendix lumen contained a fecolith, and sections showed focal mucosal ulceration with transmural inflammatory infiltrates composed predominantly of neutrophils and eosinophils, confirming acute appendicitis. The postoperative course was uneventful, and the child was discharged in stable condition.

**Figure 3:**
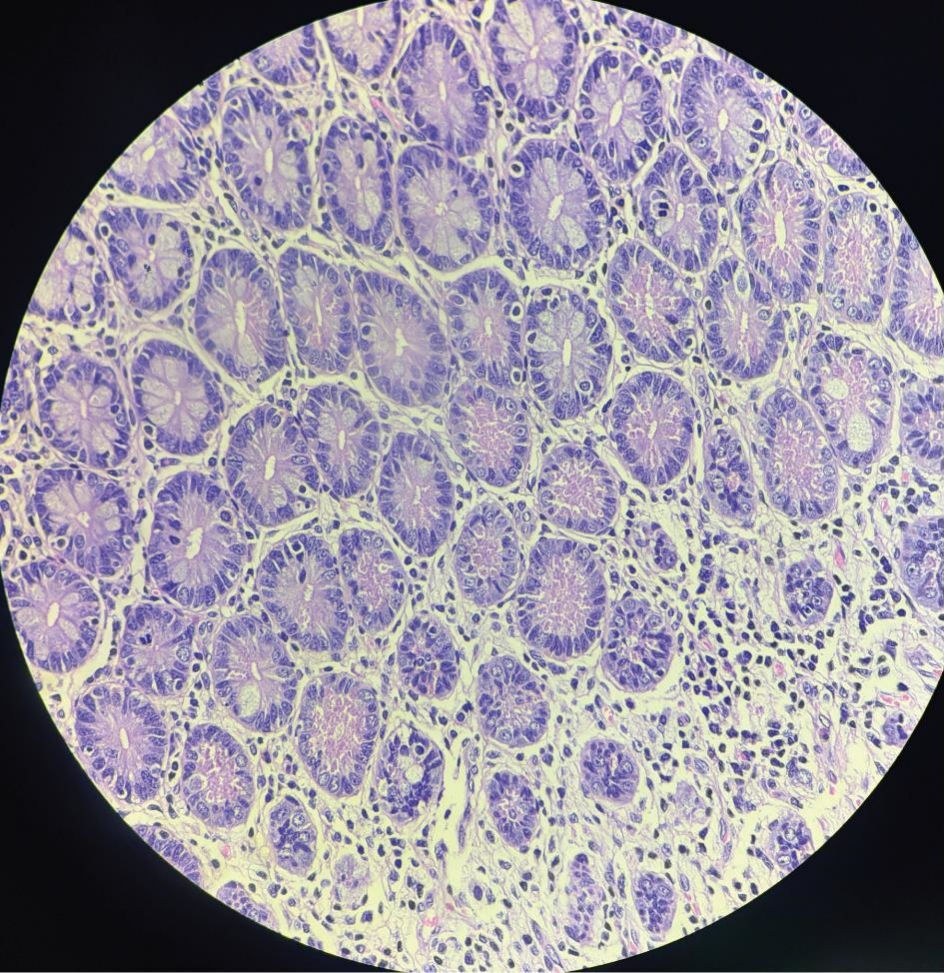
Microscopic section of Meckel’s diverticulum demonstrating focal Paneth cell metaplasia (H&E stain).

## 3. Discussion

This case is notable for three main reasons. To begin with, it illustrates the persistent diagnostic difficulty of asymptomatic Meckel’s diverticulum in children. Large reviews have shown that pediatric Meckel’s diverticulum may present with obstruction, inflammation, or bleeding, yet the diagnosis is frequently not established preoperatively because the symptoms and routine imaging findings are nonspecific [1, 2]. In the present patient, the chronicity of pain, recent vomiting, ultrasonographic identification of an appendicolith, and associated mesenteric lymphadenitis together created a plausible appendiceal diagnosis, while Meckel’s diverticulum remained unrecognized until laparoscopy. This pattern is consistent with contemporary pediatric literature showing that complicated Meckel’s diverticulum may clinically mimic appendicitis and may only become evident at the operation [3].

Furthermore, this case reinforces the practical value of diagnostic laparoscopy in children with atypical or discordant abdominal presentations. Published pediatric series have shown laparoscopy to be both safe and effective in the management of complicated Meckel’s diverticulum while also offering the important diagnostic advantage of complete abdominal evaluation through minimally invasive access [4, 5]. In this child, laparoscopy did more than confirm appendiceal disease; it prevented a missed second pathology by identifying a dilated Meckel’s diverticulum with proximal small bowel dilatation. That finding was clinically relevant because the prolonged duration of symptoms and partly obstructive pattern were not fully explained by isolated acute appendicitis alone. The case therefore supports a low threshold for systematic inspection of the distal ileum when operative findings do not adequately account for the overall symptom burden.

Finally, concomitant appendicitis and Meckel’s diverticular pathology are distinctly uncommon but increasingly recognized in case literature [6]. In our patient, the appendiceal lesion was not incidental: the appendix contained a fecolith and showed histologically confirmed acute transmural inflammation. At the same time, the dilated Meckel’s diverticulum with proximal loop dilatation suggested an additional ileal source contributing to the child’s prolonged and partly obstructive clinical course. Simultaneous treatment of both lesions was therefore justified and likely prevented the persistence of symptoms from unaddressed dual pathology.

The histopathologic finding of focal Paneth cell metaplasia adds further interest to the case, although its significance should be interpreted cautiously. Paneth cells play an important role in mucosal innate immunity and epithelial homeostasis, and Paneth cell change in altered mucosal environments has been associated with chronic inflammatory and microbiome‐related adaptation [7, 8]. However, the specific clinical relevance of focal Paneth cell metaplasia in Meckel’s diverticulum is not well established. In the present case, this finding may represent a localized mucosal response to chronic irritation or injury, but no firm pathobiologic conclusion can be drawn. Its importance therefore lies less in proving the mechanism and more in highlighting the value of careful histopathologic examination of all resected Meckel’s diverticulum specimens, particularly when the clinical presentation is prolonged or unusual.

## 4. Conclusion

Concomitant Meckel’s diverticulum and acute appendicitis should be considered in children with prolonged or atypical periumbilical abdominal pain, particularly when the clinical course is not fully explained by preoperative imaging or by appendiceal findings alone. Diagnostic laparoscopy is valuable because it enables the recognition and definitive treatment of dual pathology in a single sitting. This case also highlights that Meckel’s diverticulum may be diagnosed only intraoperatively and that histopathologic examination may reveal uncommon associated findings such as focal Paneth cell metaplasia, the clinical significance of which remains uncertain.

## Author Contributions

Aditya Pachwa contributed to the conceptualization, methodology, investigation, data curation, formal analysis, supervision, project administration, and resources. Chaitanya Laasya Reddy Arimanda contributed to conceptualization, methodology, investigation, data curation, software, and funding acquisition. Agasthya Daggu contributed to the methodology, validation, and visualization. Aditya Pachwa, Chaitanya Laasya Reddy Arimanda, and Agasthya Daggu contributed to writing the original draft.

## Funding

The authors have nothing to report.

## Disclosure

All authors contributed to reviewing and editing the manuscript and approved the final version.

## Consent

Informed consent was obtained from patients guardians.

## Conflicts of Interest

The authors declare no conflicts of interest.

## Data Availability

The data that support the findings of this study are available upon request from the corresponding author. The data are not publicly available due to privacy or ethical restrictions.
